# Reduced T Regulatory Cell Response during Acute *Plasmodium falciparum* Infection in Malian Children Co-Infected with *Schistosoma haematobium*

**DOI:** 10.1371/journal.pone.0031647

**Published:** 2012-02-14

**Authors:** Kirsten E. Lyke, Abdoulaye Dabo, Charles Arama, Modibo Daou, Issa Diarra, Amy Wang, Christopher V. Plowe, Ogobara K. Doumbo, Marcelo B. Sztein

**Affiliations:** 1 Center for Vaccine Development, University of Maryland School of Medicine, Baltimore, Maryland, United States of America; 2 Malaria Research and Training Center, University of Bamako, Bamako, Mali; 3 Department of Immunology, University of Stockholm, Stockholm, Sweden; 4 Howard Hughes Medical Institute, Baltimore, Maryland, United States of America; The George Washington University Medical Center, United States of America

## Abstract

**Background:**

Regulatory T cells (Tregs) suppress host immune responses and participate in immune homeostasis. In co-infection, secondary parasite infections may disrupt the immunologic responses induced by a pre-existing parasitic infection. We previously demonstrated that schistosomiasis-positive (SP) Malian children, aged 4–8 years, are protected against the acquisition of malaria compared to matched schistosomiasis-negative (SN) children.

**Methods and Findings:**

To determine if Tregs contribute to this protection, we performed immunologic and Treg depletion *in vitro* studies using PBMC acquired from children with and without *S. haematobium* infection followed longitudinally for the acquisition of malaria. Levels of Tregs were lower in children with dual infections compared to children with malaria alone (0.49 versus 1.37%, respectively, P = 0.004) but were similar months later, during a period with negligible malaria transmission. The increased levels of Tregs in SN subjects were associated with suppressed serum Th1 cytokine levels, as well as elevated parasitemia compared to co-infected counterparts.

**Conclusions:**

These results suggest that lower levels of Tregs in helminth-infected children correlate with altered circulating cytokine and parasitologic results which may play a partial role in mediating protection against falciparum malaria.

## Introduction

Regulatory T cells (Tregs) are a subset of CD4^+^ T cells associated with immunotolerance and appear to play an important role in modulating the host response to parasites. Co-parasitic infections such as schistosomiasis and malaria are common in endemic areas. We and others have shown *S. haematobium* infection to modestly protect against uncomplicated malaria in West African children [1], [2]. However, results are mixed when examining the effect of other helminth infections upon the acquisition of malaria with some studies suggesting a protective effect [3], [4] and others noting enhanced burden of malarial disease [5]–[7]. The mechanisms contributing to protection from, or susceptibility to, malaria remain unclear but there is evidence of immunologic perturbation in co-infection [8]–[11]. Given the role of Tregs in immune homeostasis, we hypothesize that the activity of Tregs in children infected with *S. haematobium* may be important in facilitating protection from malaria.

Tregs are characterized by high expression of CD25 [12], [13], and constitutively express transcription factor forkhead box 3 protein (FOXP3); thought to contribute to differentiation [14], [15]. Putative surface markers such as glucocorticoid-induced tumor necrosis factor receptor (GITR), cytotoxic T-lymphocyte antigen 4 (CTLA-4, CD152), and memory markers have been reported [16]–[20]. Based upon cytokine secretion patterns and cell surface markers, Tregs can be categorized into naturally occurring (nTregs) and induced/acquired Tregs. Natural T regs are defined as being FOXP3^+^ and thymus-derived, while inducible Tregs develop in the periphery from conventional CD4^+^ T cells after exposure to regulatory cytokines and other stimuli [19]. However the lineage relationships and the contribution of other Treg populations to immune homeostasis is poorly understood and complicated by the lack of definitive cell markers [21], [22].

Little is known about the Treg response to human *S. haematobium* infections. Chronic egg-laying schistosomes exert a persistent stimulatory effect on the host immune system, due in large part to egg antigens [23], [24]. Antigen-specific Treg-associated immunosuppression has been described with *S. mansoni* [25] and other helminths [26], [27]. Recently, Tregs have been suggested to positively correlate with burden of infection in malaria-negative, *S. haematobium*-positive Zimbabwean children however a comparison group of children without helminth infection was not included [28]. In schistosome-infected mice, nTregs suppress effector T cell response to egg-induced inflammation [29] while adoptive cell transfer of CD4^+^CD25^+^ T cells reduce egg-induced liver injury and enhance survival via an IL-10 pathway [30]. In summary, it appears that Tregs may ameliorate the robust host immune response induced by schistosomal egg production.

In malaria, Tregs may facilitate parasitemia and abrogate host clearance of infection. *In vivo* depletion of natural CD4^+^CD25^+^ T cells prior to infectious challenge with a lethal dose of *Plasmodium spp*. results in enhanced mouse survival and eradication of parasitemia [31], [32]. Abrogation of IL-10 and TGF-β response or depletion of CD4^+^CD25^+^ Treg cells restores the Th1 cytokine balance allowing the murine host to control and eradicate the parasitic infection [31], [33]. In humans, Treg induction has been demonstrated in adults with natural as well as experimental sporozoite-induced falciparum malaria [34], [35] and appears to correlate with increased clinical malaria [35]. Parasitemia correlates with a burst of monocyte-derived TGF-β production, increases in CD3^+^CD4^+^CD25^hi^ T cells and FOXP3 expression [34]. A functional deficit of Tregs has been suggested as one factor in the inherent protection against falciparum malaria that the Fulani possess compared to sympatric ethnic groups such as the Mossi in West Africa [36].

Limited data are available regarding Tregs in co-parasitic infected humans. Paradoxically, reduced levels of Treg memory cells were found to be present in Kenyan children co-infected with *S. mansoni* and falciparum malaria [37]. Given our findings that the presence of *S. haematobium* correlates with protection against clinical falciparum malaria in an age-specific manner in Malian children, the role of Tregs as a potential immunologic factor contributing to this phenomenon was of great interest. The complexities of conducting immunologic analysis on young children in remote areas of Africa are well known and, largely responsible for the paucity of immunologic data. In spite of these difficulties, we provide evidence that the presence of *S. haematobium* in Malian children results in lower Treg levels at the time of a malaria infection compared to children without helminthic infection.

## Methods

### Study population and clinical trial design

Bandiagara (pop. ∼13,600) is located in Mali, West Africa and has intense seasonal transmission (July-December) of *P. falciparum* malaria. The entomologic inoculation rate is 20–60 infected bites per month during peak transmission and, children experience a mean of 1.54 symptomatic malaria episodes per season [38]. *S. haematobium* and *mansoni* are endemic to the area [39], [40] with *S. haematobium* prevalence of 25% in children aged 4–14 years and ∼50% in adults [1]. This study was conducted over two sequential malaria transmission seasons (2002-03) and study details have previously been reported [1], [8]. Briefly, children aged 4–14 years of age, diagnosed as having asymptomatic *S. haematobium* (SP), were age, gender and residence-matched to a child without schistosomiasis (SN) prior to malaria transmission. Children were followed weekly over the malaria transmission season (25 weeks) and at a dry season follow-up appointment (∼9 months after enrollment at a time when standing water pools had dried and schistosoma transmission had ceased). The primary endpoint of the clinical trial was time to first clinical malaria infection. Clinic personnel were available 24 hours-a-day throughout study duration to detect, examine and treat symptomatic malaria episodes. A clinical episode of malaria was defined as *P. falciparum* parasitemia and axillary temperature ≥37.5°C on active surveillance, or parasitemia and symptoms leading to treatment-seeking behavior in the absence of other clear cause on passive surveillance. All children were pre-treated with albendazole to eliminate concomitant helminth infections and study samples were drawn at the time of their first clinical malaria episode (or at study week 25/Day 175 in the absence of a clinical infection) and again at the final dry season appointment. Children were optimally treated for schistosomiasis with praziquantel at the final appointment.

### Ethics

The trial was conducted in compliance with the Declaration of Helsinki. Study protocols were reviewed and approved by the University of Bamako's Institutional Review Board (IRB) as well as the University of Maryland IRB. Village permission to conduct research was obtained from village chiefs, government officials and traditional healers prior to study initiation. Individual written informed consent was obtained from the parent or legal guardian of each child prior to screening and enrollment. All children displaying gross hematuria or symptoms of genitourinary pathology were treated with praziquantel (40 mg/kg) therapy and discharged from the study.

### Sample Collection

Patient whole blood (5–10 mL) was collected at the study clinic into sterile eppendorf and EDTA tubes on admission, prior to institution of anti-malarial therapy, and immediately refrigerated. Sera was processed as previously described [8]. Blood was processed by density centrifugation, within two hours of acquisition, utilizing lymphocyte separation medium (ICN Biomedical Inc, Aurora, OH) following standard techniques [41]. Peripheral blood mononuclear cells (PBMC) were resuspended in media and linear-rate frozen using isopropyl alcohol containers (Nalgene, USA) to −70°C in the field site before transfer in liquid nitrogen storage containers to the University of Maryland at Baltimore. Samples for this experiment were chosen based on the availability of both a wet and dry season time point ***and the presence of at least 15.0×10^6^ cells*** for each child at each time point.

### PBMC depletion and stimulation

Thawed PBMC were rested overnight at 37°C, 5% CO_2_ and washed. A portion of cells (2.0×10^6^) was removed to serve as negative (media) and positive (stimulation with 10 µg/ml *Staphylococcus* enterotoxin B (SEB); Sigma, St. Louis, MO) controls and to measure the Treg population prior to stimulation. The remaining PBMC were split into two aliquots consisting of 3.0–4.8×10^6^ cells each and either mock-depleted or depleted of CD25^hi^ cells using Dynabead pan-mouse IgG or CD25 magnetic beads, respectively (Invitrogen, Carlsbad, California) at a bead to PBMC ratio of 5∶1. Depleted (mock and CD25^hi^) PBMC (3.0×10^6^) were stimulated with media, malaria antigen pool (consisting of Apical Membrane Antigen 1 (AMA1) [42] and Merozoite Surface Protein 1 (MSP1_42_) [43], and *S. haematobium* antigen pools (soluble egg antigen (SEA) and soluble worm antigen protein (SWAP). Antigen stimulation for the assay was optimized at 5 µg/ml/antigen. The malaria antigens chosen represent two vaccine candidates being tested at the same field site in Mali while the *S. haematobium* antigens are commonly used in schistosoma research. The 3D7 stain of malaria is well documented at the Malian site and strain specific protection against an AMA1 vaccine has been established [44]. AMA1 has been shown to elicit CMI [45]; however the effect of either malaria antigen towards Treg induction is unknown. Using an optimized protocol [46], all cells were stimulated for 2 h before protein transport was blocked by adding 0.5 µl/tube GolgiPlug (BD Pharmingen) followed by overnight incubation.

### Flow Cytometry Staining and Analysis

PBMC were stained with fluorochrome-labeled monoclonal mouse anti-human antibodies against surface antigens (CD3-Energy Coupled Dye (ECD, Beckman Coulter, clone UCHT1), CD4-allophycocyanin (APC)-Cy7 (BD Biosciences, clone SK3), CD8-Alexa700 (BD Biosciences, clone RPA-T8), CD25-phycoeryhtrin (PE)-Cy7 (BD Biosciences, clone M-A251), CD19 (InVitrogen, clone SJ25-C1)/Vivid-Pacific Blue, and CD14-biotin (InVitrogen, clone TűK4 followed by streptavidin-Pacific Orange), followed by fixation/permeabilization by using cytofix/cytoperm solution (eBiosciences) and intracellular staining with monoclonal antibodies to IFN-γ-APC (BD Biosciences, clone B27), IL-2-PE (BD Biosciences, clone MQ1-17H12), FOXP3-Alexa488 (eBiosciences, clone 236A/E7), and CD69-PE-Cy5 (Beckman Coulter, clone TP1.55.3). In selected experiments monoclonal antibodies to IL-10-APC (BD Biosciences, clone JES3-19F1) and TGF-β-PE (IQ Products, clone TB21) were also used. Cells were then resuspended in 1% formaldehyde fixation buffer and analyzed using a MoFlo flow cytometer/cell sorter system (Beckman Coulter). PBMC from healthy subjects were used as internal controls in the experiments. A total of 100,000–800,000 events (mean∼500,000) in the forward and side scatter (FS/SS) lymphocyte gate were collected per sample. List-mode data files were analyzed using WinList 6.0 3D (Verity Software House, Topsham, ME). An amine reactive dye (ViViD, Invitrogen, Oregon) was used as a dead cell discriminator and B lymphocytes (CD19^+^) were excluded from analysis. Doublets/aggregates were subtracted from analysis and gate placement determined with the aid of Fluorescence Minus One (FMO) controls. Cytokine secretion from macrophages/monocytes (CD14^+^) was analyzed separately. Specimens were included in the analysis if (1) the cell viability was >75% after thawing and (2) cells were shown to be functionally active as determined by the production of IFN-γ by at least 0.2% CD3^+^ cells after stimulation with SEB. A response was considered specific if the differential in the number of positive events between experimental (stimulated with malaria or schistosoma antigen pools) and negative control (media) cultures was significantly increased by χ^2^ analysis. CD69 has been shown to be upregulated on activated effector T cells but not Tregs [47]. T regulatory cells were therefore defined as the population of cells that were CD3^+^CD19^−^Vivid^−^CD4^+^CD25^hi^CD69^−^FOXP3^+^ [34], [47]. As Tregs ameliorate immune responses, enhanced production of cytokines (IL-2 and IFN-γ) should result upon depletion of CD25^hi^ within the stimulated PBMC population. A positive response to Treg cell depletion was defined as (1) The net percentage of cytokine producing cells was ≥0.05% in CD25^hi^ depleted cells as compared to mock depleted PBMC after subtraction of media control values; (2) the number of positive events between the CD25^hi^ depleted and mock depleted cultures was significantly increased by χ^2^ analysis; and (3) the number of positive events in the stimulant pool compared to the media control in the CD25^hi^ depleted cultures was significantly increased by χ^2^ analysis.

### Lymphocyte proliferation assay

If sufficient cells remained, 100,000–150,000 of mock- or CD25^+^-depleted cells/well were plated in triplicates, incubated with media, anti-CD3^+^/CD28^+^ beads as a positive control, malaria or schistosoma pools at 10 µg/ml for 5 days and [^3^H]-thymidine incorporation determined as described [48]. Stimulation indices (SI) were calculated by dividing the mean counts per minute (cpm) of triplicate wells post-stimulation by the mean cpm observed in media controls. Values that fell outside the 95% CI of replicate wells were excluded from analysis. Positive samples were defined as (1) mean of wells with malarial pools differing significantly from media controls by paired t-test (one-tailed, P<0.05), (2) Stimulation index (SI)>2 and, (3) mean cpm>1,000 than media control. Stimulation indices were calculated after [^3^H]-thymidine incorporation in PBMC before and after CD25^hi^-depletion and stimulation with malaria and schistosoma antigen at two time points. Results were excluded if anti-CD3/CD28 stimulated cells failed to exhibit strong response.

### Circulating cytokine measurements

Cytokine levels were determined utilizing cytometric bead array technology (BD Biosciences, San Diego, CA) and fluorescence detection by flow cytometry following the manufacturer's recommendations with modifications, as previously described [8], [49]. The results of individual cytokine levels for only the volunteers included in this study were examined. These determinations were part of a greater pool of data previously reported [8]. The lower limit of detection was determined to be 2.5–5.0 pg/ml depending upon the cytokine.

### Statistics

Statistical analysis was performed on GraphPad Prism 5 (Graphpad Software, Inc., San Diego, CA) and demographic and immunologic data were stratified and evaluated by age group (age 4–8 and 9–14 years). Student t-test (two-tailed) or Mann-Whitney U test were used to compare continuous data and χ^2^ analysis, using Mantel-Haenszel or Fisher Exact (two-tailed) as appropriate, was performed for categorical data. Pearson product-moment correlation coefficient was calculated utilizing GraphPad Prism 5. A significance level of P<0.05 was considered statistically significant.

## Results

### Sample Population

We previously found that underlying infection with *S. haematobium* offered modest but significant clinical protection against *P. falciparum* malaria in an age-specific fashion (children aged 4–8 years) to children exposed to both parasitic infections [1]. Only samples from children with ***>15.0×10^6^ PBMC/time point*** (n = 52, mean age 8.5 years) were thawed and examined. Each experiment consisted of a U.S. malaria-naïve adult control, an SP child and an age-matched SN child. Evaluative data was available from 26 SP children (18 of whom developed malaria (SP Mal) and 8 who did not acquire malaria (SP no Mal) during the transmission season) and 23 SN children, all of who developed malaria (SN Mal) (total = 49). Very few SN children remained clinically malaria-free throughout the malaria transmission season and did not have adequate cells to pursue the immunologic analyses described. Samples were excluded if the viability or thawed quantity of PBMC was insufficient (n = 3 SN children). SP Mal children had a statistically longer time to first clinical malaria infection, reduced geometric mean parasite density at the time of malaria infection, and a trend towards reduced overall amount of malaria infections detected compared to age-matched SN Mal children (Table 1). There was no age-related difference in the character of the malaria infection in SP children or the amount of schistosoma eggs excreted between SP Mal children who developed malaria and SP no Mal children in a single wet season.

**Table 1:** Demographics.

Category	Age Strata (years)	SP Mal	SN Mal	P value
**Mean age (n)**	4–14	8.4 (26)	8.5 (23)	ns
	4–8	6.3 (15)	6.4 (11)	ns
	9–14	10.8 (11)	10.9 (12)	ns
**Female (%)**	4–14	8 (30.8)	11 (47.8)	ns
**Eggs (range)** a	4–14	100 (2–1371)	0	n/a
	4–8	62 (2–296)	0	n/a
	9–14	144 (2–1371)	0	n/a
**Malaria episodes** b **(range)**	4–14	1.52 (1–4)	2.04 (1–4)	0.06
**Days to first malaria episode** b **(range)**	4–14	76 (49–113)	21.6 (3–40)	**<0.0002**
**Parasitemia** b ^**,**^ c **(range)**	4–14	4,073 (1,500–47,950)	10,237 (1,000–298,175)	**0.03**

Demographic characteristics at enrollment and features of subsequent *P. falciparum* malaria infections of *Schistosoma haematobium*-positive (SP Mal) and age-matched *S. haematobium*-negative (SN Mal) Malian children contributing PBMC for immunologic analysis.^a^

a Urinary egg excretion detected in 10 ml of filtered morning (10 am to 2 pm) urine.

b Results for children who did not develop malaria (n = 8) are not included these calculations. If no statistical difference was noted between children in the 4–8 year old category compared to the 9–14 year old category, the results were combined.

c Geometric mean parasite density per mm^3^.

### Regulatory T cells

**Figure 1:**
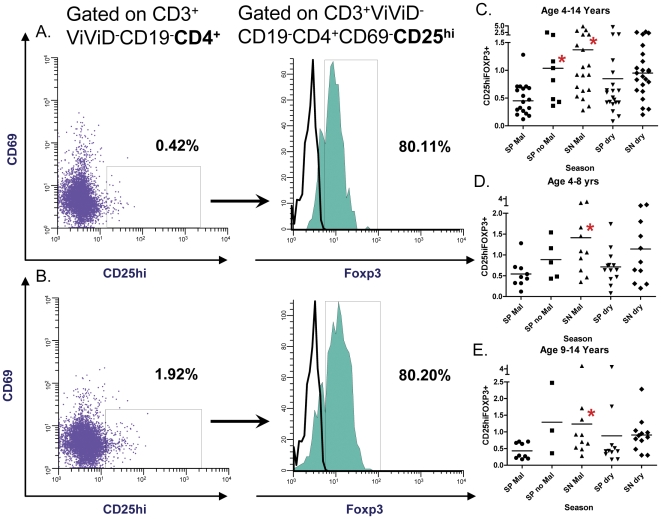
T regulatory cell detection. Quantification of T regulatory cells gated on CD3^+^ViViD^−^CD19^−^CD4^+^ with representative plots depicting the percentage of CD25^hi^ and FOXP3^+^ expression (closed histogram) in (a) a child with *S. haematobium* and concomitant *P. falciparum* malaria (SP Mal) and (b) a child with *P. falciparum* malaria (SN Mal) alone. The percentages of gated populations are denoted in each filled histogram (a, b). Region placement was aided by fluorescence minus one (FMO) determinations for CD69^+^ and FOXP3^+^ cells (open histogram). Dot plots of percentage of CD3^+^ViViD^−^CD19^−^CD4^+^CD69^−^CD25^hi^FOXP3^+^ expression and segregated by group and season are depicted in children (c) aged 4–14 years, (d) aged 4–8 years and (e) aged 9–14 years. Each dot represents one individual. Bars represent the median and asterisks depict statistically significant differences between the indicated subset of volunteers and SP children during the transmission season (paired student T test with level of significance set at P<0.05).

Paired PBMC samples obtained at two time points (malaria transmission (wet) and dry season) were examined via multiparameter flow cytometry (Figure 1, a–e). The mean total percentage of CD3^+^CD19^−^Vivid^−^CD4^+^CD25^hi^CD69^−^FOXP3^+^ cells (Tregs) were significantly higher in SN Mal children at the time of their malaria infection as compared to matched SP Mal children (1.37 (range 0.28–4.79) vs. 0.49 (0.12–1.28), P = 0.004; age 4–14 years) (Figure 1c). The U.S. malaria-naïve adult controls (n = 5) had a mean percentage of Tregs of 1.70 (range 0.88–3.84; data not shown). SP no Mal children had significantly higher mean percentages of Tregs than SP Mal children [1.12 (range 0.36–2.47) vs. 0.49 (0.23–1.28), P = 0.004]. The mean total percentage of Tregs in SN Mal children was higher than SP no Mal children but was not statistically significant (1.37 vs. 1.12, P = 0.61). The percentage of Tregs was statistically equivalent in SN children compared to SP Mal children after recovery from malaria infection (i.e., during the dry season follow-up) (1.02 (range 0.2–2.72) vs. 0.85 (0.2–1.76), P = 0.51). Results are also depicted after age stratification (Figure 1, d and e). The peripheral parasitemia was compared to the total Treg percentage utilizing Pearson product correlation (ρ = 0.315, P = 0.06).

### Intracellular Cytokine Expression to Antigenic Stimulation

#### Interleukin-2 and Interferon gamma

**Figure 2:**
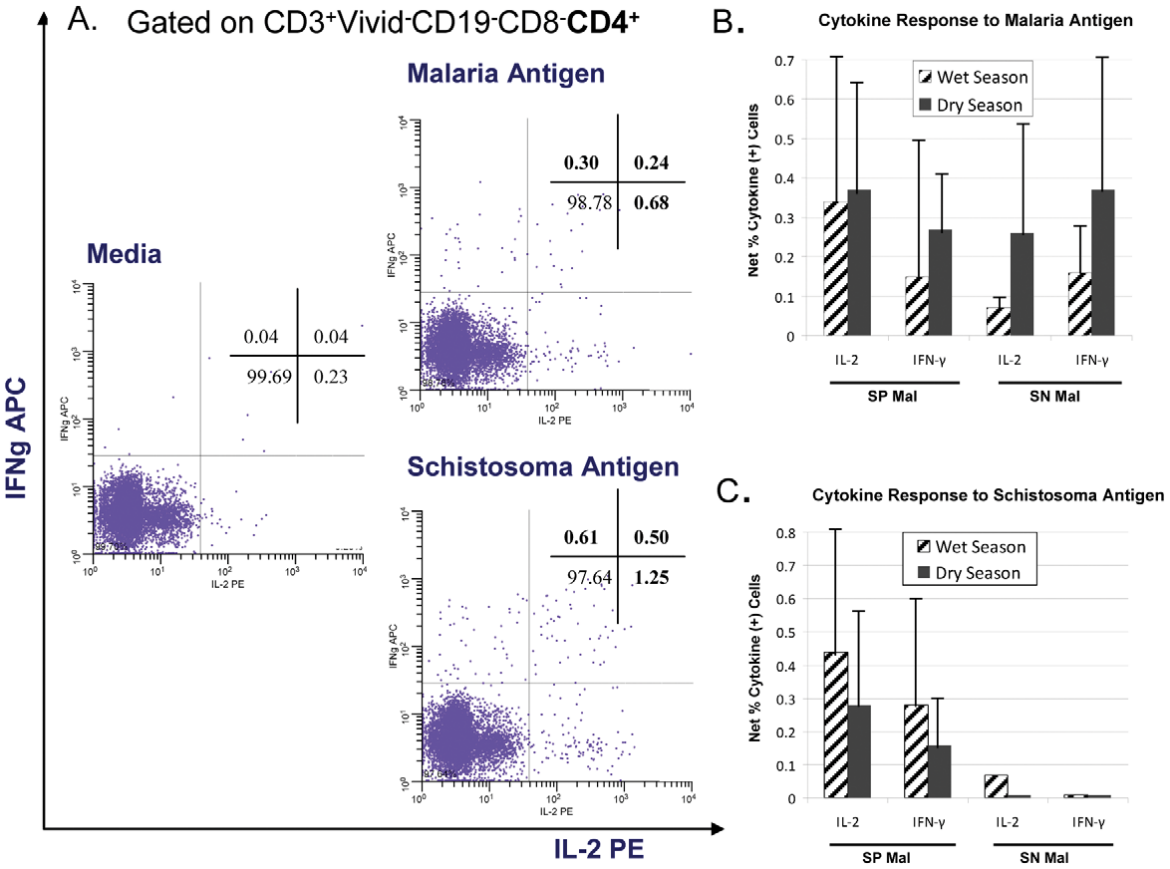
Intracellular cytokine production to malaria and schistosoma antigens. Representative example of intracellular cytokine (IL-2 and IFN-γ) detection in PBMC, from a child with concomitant *S. haematobium* and *P. falciparum* infection, in response to both malaria and schistosoma pooled antigen stimulation and media control for comparison (panel a). Mean intracellular cytokine production from PBMC stimulated with pooled (b) malaria and (c) schistosoma antigens collected in children aged 4–14 years with (SP Mal) and without S. *haematobium* (SN Mal) measured during the transmission and follow-up dry season. Cytokine-producing cells were found to be predominantly CD3^+^CD19^−^CD4^+^CD8^−^ T cells. Cytokine expression is reported as net % cytokine producing cells (stimulant minus media control) and standard deviations.

Intracellular cytokine production from PBMC stimulated with pooled malaria and schistosomal antigens was measured (Table 2 and Figure 2) in 15 SP Mal and 17 SN Mal children (n = 32). Three SP no Mal were excluded from analysis as the parameters of their clinical course did not match the children who acquired malaria. The majority of cytokine-producing cells were found to be CD3^+^CD19^−^CD4^+^CD8^−^ T cells with minimal levels (<0.03%) observed from both Tregs (CD3^+^CD19^−^CD4^+^CD8^−^CD69^−^CD25^hi^FOXP3^+^) and monocytes/macrophages (CD3^−^CD19^−^CD14^+^) (data not shown). Significant cytokine expression was measured after malaria antigen stimulation of PBMC in both SP children and SN children (Figure 2); however little difference was noted between the two groups during either the wet or the dry season (Table 2). When the results were stratified by age, differences were noted. Very little cytokine expression was measured in younger children (aged 4–8 years) after PBMC stimulation with the malaria antigen pool. Significant IL-2 expression was observed in the majority of older (9–14 years) SP Mal children as compared to age-matched SN Mal children; a finding that was also noted during the dry season with the additional finding that significantly more IFN-γ production was also measured. The net percentages of CD4^+^ T cells expressing IL-2 or IFN-γ that were statistically significant after antigenic stimulation as depicted in Figure 2B, C (due to low numbers, the results of younger and older children were combined). The net percentage of IL-2 expression was not significantly different in SP Mal children compared to SN Mal children (net mean percentage: 0.34 (range 0.04–0.98) vs. 0.07 (range 0.04–0.11), P = 0.11). A general trend towards an increased proportion of cytokine secreting cells at the dry season compared to the wet (malaria transmission) season (Figure 2b) was seen but was not significant.

**Table 2:** Intracellular Cytokine Expression to Antigen Stimulation.

			Malaria Antigen Pool^a^				Schistosoma Antigen Pool^a^			
Season	Cohort	Age (yrs)	IL-2 (%)	P^b^	IFN-γ (%)	P^b^	IL-2 (%)	P^b^	IFN-γ (%)	P^b^
Wet	SP	4–8	0/6 (0)	ns	1/6 (17)	ns	2/6 (33)	ns	2/6 (33)	ns
	SN^c^		1/5 (20)		1/5 (20)		1/5 (20)^d^		1/5 (20)^d^	
	SP	9–14	7/9 (78)	**0.02**	4/9 (44)	ns	7/9 (78)	**0.007**	6/9 (67)	**0.003**
	SN		2/10 (20)		3/10 (30)		0/10 (0)		0/10 (0)	
	SP	4–14	7/15 (47)	ns	5/15 (33)	ns	9/15 (60)	**0.002**	8/15 (53)	**0.005**
	SN		3/15 (20)		4/15 (27)		1/15 (7)		1/15 (7)	
Dry	SP	4–8	2/6 (33)	ns	1/6 (17)	ns	4/6 (67)	0.06	2/6 (33)	ns
	SN		4/5 (80)		2/5 (40)		0/5 (0)		0/5 (0)	
	SP	9–14	6/11 (55)	0.06	7/11 (64)	**0.02**	9/11 (82)	**<0.001**	7/11 (64)	**0.001**
	SN		2/12 (17)		2/12 (17)		0/12 (0)		0/12 (0)	
	SP	4–14	8/17 (47)	ns	8/17 (47)	0.16	13/17 (76)	**<0.001**	9/17 (53)	**0.0009**
	SN		6/17 (35)		4/17 (23)		0/17 (0)		0/17 (0)	

Results of intracellular cytokine expression after antigenic stimulation of PBMC acquired from age-matched children with (SP Mal) or without *S. haematobium* (SN Mal) during the malaria transmission (wet) season at the time of a malaria episode and again during a dry season follow-up visit. The numerator results represent those individuals with statistically significant increases in the percentage of cytokine expressed compared to media controls after PBMC stimulation and the denomenator represents the total number of individual PBMC samples analyzed.

a PBMC were stimulated with a malaria antigen pool (Apical Membrane antigen 1 and Merozoite Surface Protein 1) or with a *S. haematobium* pool (soluble egg antigen and soluble worm antigen protein). SP no Mal children were not included in this analysis.

b χ^2^ analysis, using Mantel-Haenszel or Fisher Exact (two-tailed) as appropriate, was performed between *S. haematobium* positive (SP Mal) vs. *S. haematobium* negative (SN Mal) children in each age category. P value significance set at <0.05. Not significant  =  ns.

c Three experiments were excluded due to insufficient or poor viability cell quantities to allow proper interpretation of data.

d IL-2 and IFN-g production was noted in one SN individual. Urine was negative for eggs both at enrollment and at dry season follow-up suggesting a false positive result.

Schistosoma antigen recognition was measured with significant IL-2 and IFN-γ expression observed after PBMC stimulation in the majority of SP children (78% malaria transmission; 82% dry season) but not in SN children (Table 2). Demonstrable IL-2 and IFN-γexpression during the wet season was seen in a majority of older SP children (aged 9–14 years) (7/9 (78%) and 6/9 (67%) respectively), which was not noted in younger children suggesting age-associated acquisition of immunity. Of those SP children with significant cytokine production to schistosoma antigen stimulation, the net percentages of cytokine expression were 0.44% (range 0.07–1.19) and 0.12% (range 0.07–1.02) for IL-2 and IFN-γ, respectively, during the wet season. The total percentage of cytokine expression was not significantly different compared to results measured during the dry season (Figure 2c). Only one SN child was noted to have detectable IL-2 production after schistisoma antigen stimulation of PBMC acquired during the wet season. This 8 year female child had no demonstrable urinary egg excretion in 3 sequential urine samples prior to the malaria transmission season and had no evidence of eggs in the urine at her dry season follow-up. Furthermore, the flow cytometric analysis on PBMC acquired during the dry season did not demonstrate enhanced IL-2 production suggesting that the result may represent a false positive. Malaria-naïve U.S. adult controls had no detectable increase of intracellular cytokines to either antigen stimulant.

### Transforming Growth Factor beta and Interleukin 10 Expression

In selected experiments we examined TGF-β and IL-10 intracellular production after malaria and schistosomal antigen stimulation in PBMC obtained from 8 SP children (4 SP Mal and 4 SP no Mal) and 6 SN Mal children with malaria, during the wet and dry season follow-up. Cytokine expression was examined in cells gated on CD3^+^CD4^+^CD25^hi^FOXP3^+^ (Tregs) after antigen stimulation and, although the numbers were low, it did establish that detectable cytokine could be measured in these cells (Table S1). Measureable TGF-β levels were noted after malaria antigenic stimulation in 50–75% of PBMC in all cohorts during the wet season and dry season; however the mean percentage of expression was diminished by the dry season time point. Modest expression of IL-10 was noted during the wet season but negligible amounts were detected at follow-up. Only children with detectable *S. haematobium* expressed measureable TGF-β after antigenic stimulation of PBMC with schistosoma antigens, while IL-10 expression was minimal. Malaria-naïve U.S. adult controls had no detectable increases in expression of TGF-β or IL-10 from Tregs following exposure to either antigen.

### Depletion Studies

#### Multiparameter flow cytometry with intracellular staining

**Figure 3:**
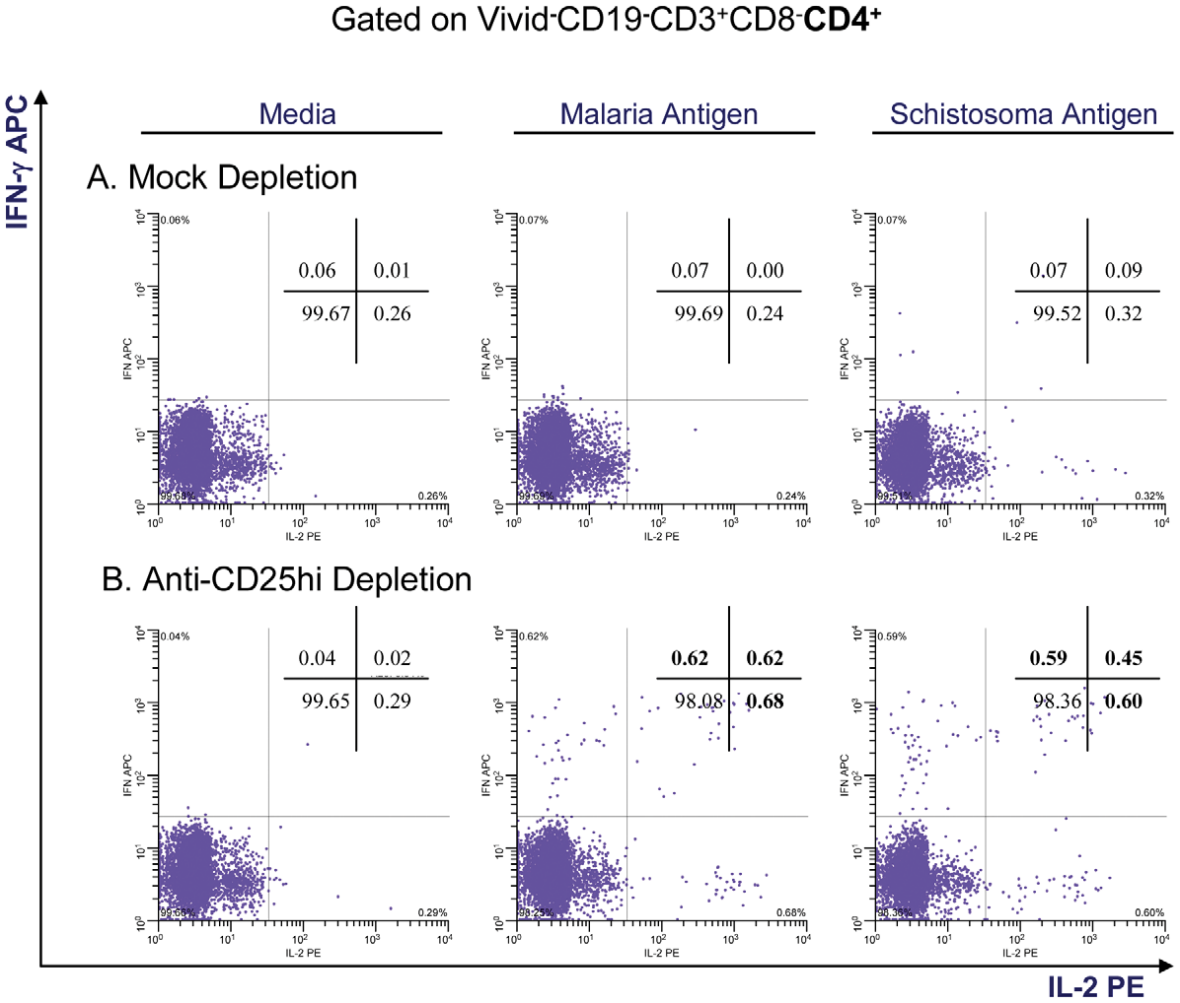
Intracellular cytokine production to malaria and schistosoma antigens after T regulatory cell depletion. Representative example of intracellular cytokine detection in PBMC, from a child with concomitant *S. haematobium* and *P. falciparum* infection, mock or anti-CD25^hi^ depleted prior to stimulation with malaria or schistosoma antigenic pools. Removal of regulatory T cells appears to reverse suppressed immunologic responses resulting in enhanced intracellular IL-2 and IFN-γ in the anti-CD25hi depleted cell population. Media controls are included for comparison.

In order to evaluate the effect of Tregs (i.e., CD25^hi^ cells) on suppression of CD4^+^ T cell cytokine expression, equal aliquots of PBMC (n = 32 individuals) were mock-depleted or depleted of CD25^hi^ cells and then stimulated with malaria or schistosoma antigen pools (n = 32). Cytokine expression was then measured after depletion of CD25^hi^ cells (compared to the mock-depleted populations) to determine if cytokine production was increased. The effectiveness of depletion was systematically evaluated by flow cytometry following treatment. Depletion resulted in removal of 90–95% of detectable CD25^hi^. The results are summarized and a representative experiment is shown in Table 3 and Figure 3.

**Table 3:** CD25^hi^ Depletion Assay Results.

		Malaria Antigen Pool^b^				Schistosoma Antigen Pool^b^			
Cohort^a^	Season	IL-2 (%)	Mean %^c^ (range)	IFN-γ (%)	Mean %^c^ (range)	IL-2 (%)	Mean %^c^ (range)	IFN-γ (%)	Mean %^c^ (range)
SP no Mal	Wet	0/3 (0)	-	0/3 (0)	-	0/3 (0)	-	1/3 (33)	-
	Dry	0/3 (0)	-	0/3 (0)	-	0/3 (0)	-	0/3 (0)	-
SP Mal	Wet	4/15 (27)	0.51(0.31–0.83)	6/15 (40)	0.27 (0.05–1.0)	2/15 (13)	0.39 (0.17–0.61)	3/15 (20)	0.39 (0.08–0.84)
	Dry	4/14 (29)	0.3 (0.05–0.57)	7/14 (50)	0.13 (0.05–0.49)	3/14 (21)	0.27 (0.05–0.72)	3/14 (21)	0.30 (0.05–0.77)
SN Mal	Wet	5/13 (38)	0.3 (0.05–1.23)	4/13 (31)	0.21 (0.05–0.56)	n/a	-	n/a	-
	Dry	6/14 (43)	0.21 (0.05–0.56)	4/14 (29)	0.16 (0.13–0.23)	n/a	-	n/a	-

Results of enhanced cytokine expression to antigenic stimulation after CD25^hi^ depletion of PBMC followed by antigenic stimulation in comparison to mock-depleted populations. The numerator represents the number of children with enhanced Interleukin 2 (IL-2) or Interferon-gamma (IFN-γ) expression after depletion compared to mock-depletion. The denominator is the number of children examined. The net percentage of cytokine expressed in CD3^+^CD4^+^CD8^−^ T cells (i.e., depletion results minus mock-depletion results) after depletion as measured by multiparameter flow cytometry is also depicted in those experiments with significant cytokine expression increase. Cells were acquired from age-matched children aged 4–14 years with or without *S. haematobium* during the malaria transmission (wet) season and again during a dry season follow-up visit. Also depicted are results from SP children who did not acquire malaria (SP no Mal) during the wet season.

a Stratification between younger children aged 4–8 years and older children aged 9–14 years revealed no significant difference so results are reported as a combined age cohort of children aged 4–14 years.

b PBMC were stimulated with a *P. falciparum* antigen pool (Apical Membrane antigen 1 and Merozoite Surface Protein 1) or with a *S. haematobium* antigen pool (soluble egg antigen and soluble worm antigen protein).

c The mean percentage increase represents the average of the net increase in cytokine production (stimulant wells minus media wells) of those experiments with statistically significant results. Significance is defined by 1) net percentage of cytokine producing cells ≥0.05% in CD25hi-depleted compared to mock-depleted PBMC, 2) the number of positive events between depleted and mock-depleted cultures was significant by Chi-square analysis and 3) the number of positive events in the stimulant pool compared to the media control in depleted cultures was significantly increased.

After PBMC from wet season samples were depleted of CD25^hi^ cells and stimulated with pooled malaria antigen, increased IL-2 and IFN-γ expression was noted in 27–43% of experiments compared to the mock-depleted cells. This finding was noted in both SP Mal and SN Mal children and across both age groups (however low numbers preclude definitive statements on statistical significance). Similarly, increased cytokine expression (29–50%) was noted in PBMC from the dry season time-point when the children had recovered from active malaria infection. The PBMC from children with enhanced IL-2 production after CD25^hi^ depletion, with a few exceptions, demonstrated enhanced IFN-γ production in response to depletion. Likewise, PBMC from children with increased cytokine production after CD25^hi^ depletion during the wet season tended to demonstrate the same findings at the dry season time point. The net percentage of cytokine increases were measured and there were no significant differences between any one group or season. No responses were observed in the SP no Mal children during the malaria transmission season (n = 3) or in the U.S. adult malaria-naïve controls.

Increased cytokine expression (compared to mock-depleted PBMC) was also measured after CD25^hi^ depletion of PBMC collected from SP Mal children and stimulated with schistosoma antigen (Table 3, Figure 3). However, no differences were noted in either the number of volunteers with increased cytokine production or in the mean net percentage increase of cytokine between the wet and dry seasons. Marginal responses were noted in SP no Mal children; however low numbers preclude definitive conclusions. No responses were observed in either the SN children or in the U.S. adult malaria-naïve controls.

We also examined the Treg percentages in those individuals with enhanced cytokine production after CD25^hi^ depletion (responders). No difference was noted between responders and non-responders. However, the mean percentage of Tregs during the malaria transmission season in SN Mal responders was significantly higher than in SP Mal responders (1.19% (range 0.28–4.79) vs. 0.35% (range 0.12–0.68), P = 0.004 Mann-Whitney U test), which mirrored the findings reported for the groups at large. The mean percentage of Tregs during the wet season for SP Mal responders was the same as in the dry season (0.35% compared to 0.41% (range 0.28–0.63) in the same individuals. Conversely, nearly every SN Mal child responder had higher Tregs percentage at the time of malaria, as compared to the dry season (1.19 (0.28–4.79) vs. 0.60% (range 0.2–0.98), P = 0.07).

#### Lymphoproliferative Responses

**Figure 4:**
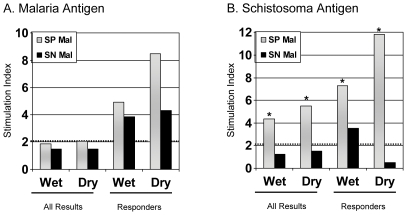
Proliferative responses to malaria and schistosoma antigens. Shown are the geometric mean lymphocyte proliferative responses as determined by [^3^H]-thymidine incorporation to malaria and schistosoma antigen pool stimulation (4a and b) in PBMC collected during the malaria transmission season and again in the dry season in children with (SP Mal, n = 20) and children without (SN Mal, n = 17) *S. haematobium*. Responders refer to those individuals with significant proliferative responses to malaria antigen stimulation (n = 8 SP Mal and n = 6 SN Mal, Figure 4a) or to schistosoma antigen stimulation during the transmission season (n = 13 SP Mal and n = 1 SN Mal, Figure 4b), with their subsequent dry season values depicted. The dotted line depicts the minimally significant SI of 2.0. The asterix depicts significant differences between SP Mal and SN Mal (P<0.001). No differences were noted between mock or anti-CD25^hi^ depletion experiments (not shown).

Of the 26 SP and 23 SN samples, results were available from 20 SP (12 aged 4–8 years and 8 aged 9–14 years) and 17 SN (11 aged 4–8 years and 7 aged 9–14 years). Results from 6 SP and 6 SN samples were excluded from analysis due to insufficient cells or inadequate proliferative response to positive control stimulation. The geometric mean stimulation indices (SI) for the groups as a whole were quite low in response to malaria antigen pools (Figure 4a). Eight of the SP (40%) and 6 of the SN (35%) children had proliferative responses (i.e., SI≥2) to malaria antigen stimulation (responders). No differences were noted in proliferative responses to malaria antigenic pools between the children with and without *S. haematobium* or between samples evaluated during the transmission season and again at a dry season time point. No differences were noted in SI between mock-depleted PBMC and anti-CD25^hi^-depleted PBMC after stimulation with malaria antigen (data not shown).

A significant proportion of PBMC collected from SP children during the wet season proliferated after schistosoma antigen stimulation compared to SN children (13/20 vs. 1/17, p = 0.0002, χ^2^ analysis) (Figure 4b). Schistosome-specific immunity appeared to be higher in the dry season. An isolated SI of 3.5 was measured in an SN child during the transmission season however PBMC from the dry season did not proliferate suggesting a false positive result (Table 2). This was not the same child noted to have intracellular cytokine production to schistosoma antigen stimulation. Again, depletion of CD25^hi^ PBMC did not result in enhanced proliferative responses at either time point (data not shown).

### Circulating Serum Cytokine Results

Circulating cytokine values were available from prior studies [8]. Data were available for all SP and SN individuals examined by flow cytometric analysis (Table S2). The mean IFN-γ and IL-2 (representative Th1 cytokines) was significantly reduced in SN children compared to SP children. Although not statistically significant, reduced IL-12p70 and IL-6 were similarly noted in SN children. This trend mirrored the prior analysis. No difference was noted in either IL-4 or in IL-10 levels (representative Th2 cytokines) between the two groups.

## Discussion

Very little is known regarding the activity of Tregs in individual human parasitic infections or the immunologic perturbations that one parasitic infection has upon the immune responsiveness to another infection in the host. We report on the presence of Tregs in both malaria and *S. haematobium* infections and measure antigen-specific cytokine production in response to malaria and schistosoma antigen stimulation of PBMC acquired from Malian children. While the limited data published to date suggests that Tregs are elevated in acute malaria, the effect of *S*. *haematobium* is unknown. Our studies in Malian children provide strong evidence that the presence of Tregs at the time of an acute falciparum malaria infection is paradoxically reduced in the presence of an underlying schistosoma infection compared to age-matched children without helminth infection. Indirect evidence supports this paradoxical finding in that children with higher percentages of Treg (SN Mal) have 1) reduced circulating Th1 (IFN-γ and IL-2) serum cytokine levels suggesting effector T cell suppression; and 2) elevated parasitemia levels compared to their matched co-infected (SP Mal) counterparts. We did not see marked differences in Th2 cytokines (e.g., IL-4, IL-10) which have been shown to be produced by inducible Tregs and limit malaria pathogenicity [50]. We did not observe an elevation in Tregs as one might expect to a helminth infection. Surprisingly little is known about Tregs in children with *S. haematobium*. While a recent study reports measuring Treg in children with *S. haematobium*, a comparison group of children without schistosomiasis was not included [28]. *S. haematobium* infection may not stimulate marked Treg elevation. The Treg differences measured between SP Mal and SN Mal children disappear after the malaria infection had been treated.

A signature role of T regulatory cells is to suppress CD4^+^ T cells [51] resulting in reduced inflammatory responses to a variety of parasites [19], [52]–[55]. We reasoned that removal of Tregs should result in enhanced intracellular cytokine expression to antigen stimulation. Increased expression of IL-2 and/or IFN-γ was noted in 30–50% of CD25^hi^-depleted PBMC acquired from SP Mal children at the time of a malaria infection, however this was also noted in SN Mal children, as well as in a proportion of samples acquired during the dry season follow up period when the children did not have active clinical malaria infection. This finding suggests that Tregs play a partial role in suppressing the immune response to antigens in a non-specific manner. It is possible that Tregs are stimulated to increase in response to malaria infection and that schistosoma co-infection exerts a down-regulatory effect on the generation of Tregs rather than affecting Treg function per se. In this case, the removal of any measurable amount of Treg followed by cell stimulation at any time point might result in a detectable increase in cytokine expression. It would be important to confirm this hypothesis by longitudinally following a cohort of children prior to the development of the malaria infection and after disease resolution. However, in this trial, we were limited to collecting small blood volumes in ill children and requiring that specimens meet high cell numbers and viability threshold at two time points. The complicated logistics of obtaining PBMC from the large cohort of children at enrollment precluded the collection of samples from being drawn prior to the malaria transmission season.

The effect that a reduction in measurable Tregs in co-infected children has on clinical malaria remains speculative. In malaria, Treg-mediated suppression of Th1 responses may aid *Plasmodium spp*. to escape from host immune defenses resulting in higher parasitemias but ultimately protecting against pathogenic responses such as cerebral malaria [31], [56]. Our paradoxical findings that Tregs are reduced in co-infected children, perhaps analogous to the reduced production of Tregs noted in the West African Fulani [36], may be of importance in explaining why children with schistosomiasis were protected from uncomplicated malaria in our prospective cohort trial. Similar to this trial, CD25^hi^-depletion did not lead to increased proliferative responses to malaria antigen in our assays [28], whereas other human studies have described increased proliferative responses in both severe and uncomplicated malaria cases [34], [57]. The Treg percentage of SN children at the time of malaria was modestly but not significantly elevated compared to dry season results possibly due to malaria-induced Treg elevation. We did not detect clinical differences in malaria signs or symptoms between SP Mal and SN Mal children as a result of these Treg differences.

There are a few issues in the present study, which deserve further investigation. We have previously detected sub-patent, asymptomatic infection during the dry season in children at this site (unpublished data) but we did not examine dry season samples for low-level parasitemia as an explanation for why Tregs may continue to exert suppressive effects on effector responses. Moreover, we are limited to observations seen to only two malaria antigens tested. MSP-1 and AMA-1 are being explored as vaccine candidates in our population and we have extensive experience with the immunologic responses to these proteins but they represent a fraction of the malaria protein repertoire. Parasitized RBCs have been found, subsequent to our analysis, to elicit two populations of Tregs expressing different amounts of FOXP3 and may have provided additional interesting results [58]. It remains unclear why younger children with schistosomiasis demonstrated greater clinical protection against malaria [1]. Variable malaria exposure undoubtedly occurs in our population of 4–14 year old children. Is the reduction of Tregs seen in SP children contributory towards a protective effect of helminths on the acquisition of malaria or is it simply an effect related to competing co-infections, possibly related to immunologic exhaustion or suppression of Treg recruitment and/or expansion? We did note that older SP children demonstrated enhanced immunologic recognition to malaria antigen compared to their age-matched SN counterparts but this is difficult to interpret in light of our clinical findings. A longitudinal study with Treg analysis done prior to, and periodically during, the malaria transmission season is planned to directly address these important unanswered questions.

The definition of what constitutes a Treg cell is a matter of some debate. While CD25 has been thought to be a definitive marker of Tregs [12], [13], others suggest that CD25, which is upregulated in activated CD4^+^ T cells, is heterogenous in Tregs [57]. Thus, we focused on a cell subset from the CD4^+^CD25^hi^ population by removing from analyses CD69^+^ cells (a marker of cell activation) [47] and corroborating that at least 80% of these cells co-expressed FOXP3, which allowed us to standardize results across many experiments by eliminating the confounding effects of heterogeneous FOXP3 expression by CD4^+^CD25^hi^ cells. Given that the percentage of FOXP3^+^ expression within CD25^hi^ cells can vary widely (as low as 10% in some estimates) [57] we feel that our approach was highly conservative with respect to defining Treg populations. It is also true that CD4^+^FOXP3^+^ cells have variable levels of CD25 expression [59], and the freeze thaw process may differentially affect cells and alter levels of CD25 expression. We also recognize that depletion beads bind only to CD25^hi^ cells which may result in Tregs remaining after depletion and fail to eliminate Tregs that are FOXP3^+^ alone (16–35% by some estimates). Thus, results may be underestimated. This may partially explain why Treg results vary widely in previous studies. In controlled experiments involving experimental malaria in naïve adults, TGF-β production correlated with parasitemia and Tregs elevation; however this finding was only noted in 50% of volunteers [34] without explanation as to why the other volunteers did not mount a Treg response. We also found that depletion studies resulted in enhanced cytokine production in approximately one third to one half of experiments without a clear rationale for the negative results. Clearly, the complexities of Treg induction in the human immune response to malaria are poorly understood.

In summary, our data provides evidence of a reduction in Tregs during acute falciparum malaria infections in schistsomiasis-infected children. Children with falciparum malaria alone had higher Tregs percentages along with reduced Th1 circulating cytokine and higher parasitemias compared to age-matched children with dual malaria and schistosoma infections. These Th1 cytokines may act to control parasitemia in the short-run while contributing to malaria pathogenicity over time. Whether the reduction of Tregs in the co-infected children mediates protection against acute falciparum malaria remains unclear. Future studies are needed to further dissect the complexities of Treg heterogeneity and to determine if the levels of Tregs in schistosoma-infected children at the beginning of the malaria transmission season contribute to malaria protection over time.

## Supporting Information

Table S1 **Cytokine Assay Results.** Results of enhanced cytokine expression after antigenic stimulation of PBMC acquired in the malaria transmission (wet) and dry seasons. The numerator represents the number of children with significant enhancement of TGF-β or IL-10 expression after pooled malaria (AMA1 and MSP1) or schistosoma antigen (SEA and SWAP) stimulation. The denominator represent the number of children examined. The net percentage of cytokine expressed in CD3^+^CD4^+^CD8^−^ T cells, as measured by multiparameter flow cytometry, is also depicted in those experiments with significant increases. Cells were acquired from age-matched children 4–14 years old with or without *S. haematobium* infection. Also depicted are results from SP children who did not acquire malaria (SP no Mal) during the wet season.
(DOC)

Table S2 **Serologic Cytokine Levels.** Serologic cytokine levels expressed in pg/ml during the first clinical malaria episode of the transmission season between Malian children aged 4–14 years with (SP Mal) or without *S. haematobium* (SN Mal).
(DOC)
