# Supplementary material for: Reduced T Regulatory Cell Response during Acute Plasmodium falciparum Infection in Malian Children Co-Infected with Schistosoma haematobium
Source: PLoS One. 2012 Feb 14;7(2):e31647. doi: 10.1371/journal.pone.0031647 (PMC3279404; doi:10.1371/journal.pone.0031647)
Supplement: Table S1 — Cytokine Assay Results. Results of enhanced cytokine expression after antigenic stimulation of PBMC acquired in the malaria transmission (wet) and dry seasons. The numerator represents the number of children with significant enhancement of TGF-β or IL-10 expression after pooled malaria (AMA1 and MSP1) or schistosoma antigen (SEA and SWAP) stimulation. The denominator represent the number of children examined. The net percentage of cytokine expressed in CD3+CD4+CD8− T cells, as measured by multiparameter flow cytometry, is also depicted in those experiments with significant increases. Cells were acquired from age-matched children 4–14 years old with or without S. haematobium infection. Also depicted are results from SP children who did not acquire malaria (SP no Mal) during the wet season. (DOC) [file pone.0031647.s001.doc]

**Table S1: Cytokine Assay Results.**

|  |  | **Malaria Antigen Pool** | | | | **Schistosoma Antigen Pool** | | | |
| --- | --- | --- | --- | --- | --- | --- | --- | --- | --- |
| **Cohort ^a^** | **Season** | **TGF-β (%)** | **Mean %**^b^ **(range)** | **IL-10 (%)** | **Mean %**^b^ **(range)** | **TGF-β (%)** | **Mean %**^b^ **(range)** | **IL-10 (%)** | **Mean %**^b^ **(range)** |
| SP no Mal | Wet | 2/4 (50) | 0.14 (0.14) | 0/4 (0) | <0.05 | 2/4 (50) | 0.05 (0.05-0.06) | 0/4 (0) | <0.05 |
|  | Dry | 0/4 (0) | <0.03 | 0/4 (0) | <0.05 | 0/4 (0) | <0.05 | 0/4 (0) | <0.05 |
| SP Mal | Wet | 3/4 (75) | 0.37 (0.07-1.01) | 2/4 (50) | 0.13 (0.09-0.16) | 3/4 (75) | 0.39 (0.17-0.61) | 1/4 (25) | 0.07 |
|  | Dry | 3/4 (75) | 0.12 (0.07-0.21) | 1/4 (0) | 0.05 | 2/4 (50) | 0.10 (0.05-0.15) | 1/4 (0) | 0.08 |
| SN Mal | Wet | 4/6 (67) | 0.57 (0.26-0.94) | 2/6 (33) | 0.16 (0.08-0.24) | 0/6 (0) | <0.05 | 0/6 (0) | <0.05 |
|  | Dry | 4/6 (67) | 0.35 (0.04-0.92) | 0/6 (0) | <0.05 | 0/6 (0) | <0.05 | 0/6 (0) | <0.05 |

**^a^** Due to the limited numbers of subjects examined, the results are reported as a combined age cohort of children aged 4-14 years.

**^b^** The mean percentage increase represents the average of the net increases in cytokine production (stimulant wells minus media wells) of those experiments with statistically significant results. Significance is defined as: 1) net percentage of cytokine producing cells > 0.05% and 2) the difference in the number of cytokine producing cells in the stimulant pool compared to the media control was significant by Chi-square analysis
