# Supplementary material for: Reduced T Regulatory Cell Response during Acute Plasmodium falciparum Infection in Malian Children Co-Infected with Schistosoma haematobium
Source: PLoS One. 2012 Feb 14;7(2):e31647. doi: 10.1371/journal.pone.0031647 (PMC3279404; doi:10.1371/journal.pone.0031647)
Supplement: Table S2 — Serologic Cytokine Levels. Serologic cytokine levels expressed in pg/ml during the first clinical malaria episode of the transmission season between Malian children aged 4–14 years with (SP Mal) or without S. haematobium (SN Mal). (DOC) [file pone.0031647.s002.doc]

**Table S2: Serologic Cytokine Levels.**

| Cohort | IFN-γ | IL-2 | IL-4 | IL-6 | IL-10 | IL-12 |
| --- | --- | --- | --- | --- | --- | --- |
| SP Mal (n=18)^a^ | | | | | | |
| Mean | 16.4 | 11.1 | 27.2 | 676 | 288 | 119.2 |
| Median | 9.9 | 8.4 | 21.3 | 152.5 | 108 | 19.5 |
| Range | 2.5-83.0 | 2.5-75.8 | 5.0-162.5 | 10.7-5,040 | 5.4-1,435 | 2.5-1,742 |
| SN Mal (n=23) | | | | | | |
| Mean | 10.3 | 3.5 | 27.2 | 176 | 388 | 61.9 |
| Median | 2.5 | 2.5 | 23.6 | 100 | 159 | 2.5 |
| Range | 2.5-43.1 | 2.5-13.1 | 5.0-72.5 | 2.5-1,123 | 10-3,341 | 2.5-421 |
| P value **^b^** | **0.04** | **<0.0001** | 0.67 | 0.06 | 0.63 | 0.16 |

^a^ SP Mal sample size include only those children with an episode of malaria

^b^ P value calculated utilizing Mann-Whitney U test for values not normally distributed
